# Computational visual ecology in the pelagic realm

**DOI:** 10.1098/rstb.2013.0038

**Published:** 2014-02-19

**Authors:** Dan-E. Nilsson, Eric Warrant, Sönke Johnsen

**Affiliations:** 1 The Lund Vision Group, Department of Biology, Lund University, 22362 Lund, Sweden; 2 Department of Biology, Duke University, Durham, NC 27708, USA

**Keywords:** visual ecology, pelagic, visual range, computational

## Abstract

Visual performance and visual interactions in pelagic animals are notoriously hard to investigate because of our restricted access to the habitat. The pelagic visual world is also dramatically different from benthic or terrestrial habitats, and our intuition is less helpful in understanding vision in unfamiliar environments. Here, we develop a computational approach to investigate visual ecology in the pelagic realm. Using information on eye size, key retinal properties, optical properties of the water and radiance, we develop expressions for calculating the visual range for detection of important types of pelagic targets. We also briefly apply the computations to a number of central questions in pelagic visual ecology, such as the relationship between eye size and visual performance, the maximum depth at which daylight is useful for vision, visual range relations between prey and predators, counter-illumination and the importance of various aspects of retinal physiology. We also argue that our present addition to computational visual ecology can be developed further, and that a computational approach offers plenty of unused potential for investigations of visual ecology in both aquatic and terrestrial habitats.

## 1. Introduction

For most terrestrial habitats, we have an intuitive feeling about the roles that vision potentially may have, and it is often possible to access the habitats for measurement and experiments. In the pelagic world, this is typically not the case. The visual world of pelagic animals is fundamentally different from our own world. In the open water and away from the surface, there are no stationary structures and no landmarks. The background of inanimate structures that so totally dominates most terrestrial visual scenes is absent in the pelagic realm. Instead, the vertical radiance distribution follows the observer, with the highest radiance seen straight upward and the lowest in the opposite direction [1]. Any objects that are visible against this background are possibly potential predators, mates or prey.

All but the upper part of the pelagic habitat is largely inaccessible to us, and pelagic animals are notoriously hard to keep in culture [2]. Behavioural and physiological experiments on these animals are either extremely challenging or impossible, but modelling of vision is comparatively tractable in the pelagic habitat because the visual background is simple and predictable. In this paper, we take the modelling approach to learn about visual ecology in the otherwise largely inaccessible pelagic realm. Our basic question is: what can animals see down there? The answer of course depends on a number of factors involving both the environment and the visual system. To be able to calculate what animals can see, it is first necessary to determine the nature of the visual task. A major choice is between pattern discrimination and object detection.

In the pelagic habitat, maximum detection range is a particularly relevant measure of visual performance [3]. Unlike vision in terrestrial habitats, where the detection range may be enormous or effectively infinite (we can see the stars), vision in aquatic habitats is severely limited by absorption and scattering of the water [3,4]. But water quality alone does not determine the range of underwater vision. It also depends on the target, the amount of daylight and the visual performance of the observer [5,6]. With good estimates of these properties, it is in principle possible to calculate the maximum range at which a target can be detected (the range also defines the equally important volume of visualization space around the animal). Obviously, the type of target must first be determined.

Bioluminescent point-sources are one obviously relevant target category, and extended opaque objects are another. But both these categories can be subdivided depending on the visual background. In the presence of any ambient light, detection of bioluminescent point sources is of different ranges depending on whether light is emitted by a transparent planktonic organism or by photophores on a non-transparent animal. Likewise, an extended black object may be covered by numerous unresolved point sources (animal with photophores) or just be a cloud of unresolved point sources on a transparent background (bioluminescent plankton in the wake behind a moving animal). To simplify calculations and still allow modelling of as many real objects as possible, we have chosen to calculate the detection range of both point sources and extended objects. For point sources, we distinguish between those seen on a black or transparent background. For detection of extended objects, we assume that they are either black or transparent and may contain point sources within their silhouette. To be visible, an extended transparent object must of course contain luminous points, for example a cloud of transparent plankton triggered to emit light in the wake of a swimming animal. Each of these four specified cases requires their own mathematical treatment.

Detection of an object against a background is a matter of comparing the signal from a visual pixel pointing at the object and a neighbouring pixel just viewing the background. In either case, light in the line of sight is constantly being lost owing to absorption and scattering, and new light is also scattered into the line of sight [3]. The original light emitted or reflected by a target is thus gradually lost and replaced. At a sufficiently long distance, the target can no longer be discriminated from the background. At exactly which distance this happens is a matter of how small contrasts the eye is able to discriminate. Ultimately, it is thus a discrimination task, which depends on the number of photons detected in each pixel and also the amount of intrinsic receptor noise (the number of false photons) contributing to the photoreceptor signals.

Even though a calculation of visual range is fundamentally straightforward, there are a significant number of factors involved in computing the photon counts [7–9]. Some of these factors can be accurately estimated but others are difficult to measure with good precision. The sheer number of parameters also implies that errors can accumulate and result in poor accuracy. For general theoretical modelling of pelagic visual ecology, this is however unlikely to be a major problem, but prediction of detection ranges in specific cases may be associated with considerable errors if many of the input parameters are inaccurate. As we show, modelling can also point to parameters that are particularly critical, and thus deserve more careful measurement.

In this paper, we present the detailed derivation of equations for calculating the range of vision for camera-type eyes spotting both point sources and extended objects. Parts of the theory were recently used to assess the significance of very large eyes in giant and colossal squid [10,11]. The treatment in this paper is more general and intended as a reference for future modelling. To demonstrate the power of this theoretical approach to pelagic visual ecology, we apply the equations to a number of different general questions relating to the significance of eye size, depth in the sea and water quality. We pay special attention to ecologically important questions such as the depth limit for the use of daylight, prey/predator relations and counter-illumination. Our aim in this paper is not to exhaust the potential of the theory, but rather to make a broad survey of questions where it may be an important tool.

## 2. Derivation of theory

### (a) Discrimination criteria

**Figure 1.**
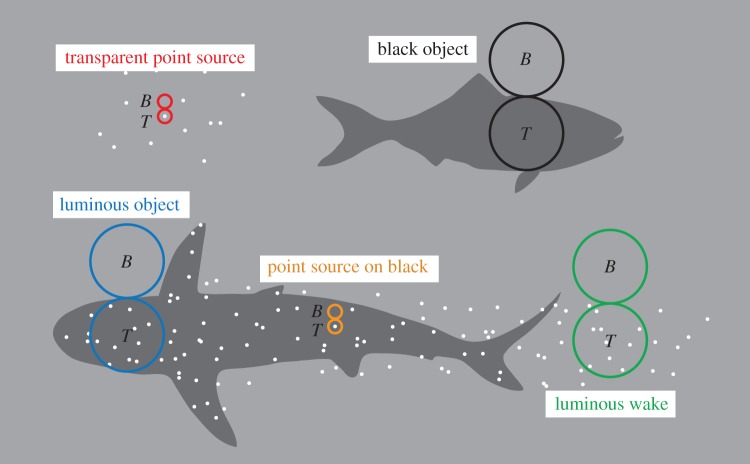
Different types of targets and detection principles on which the theoretical models are based. All detection is assumed to be discrimination of the signal from two adjacent image channels (pixels), one of which is a target pixel (*T*) and the other is a background pixel (*B*). These pixels are narrow (set by photoreceptor diameter) for point source detection, and as wide as the target for extended source detection (by dynamic pooling of circular fields of retinal photoreceptors). We model two types of bioluminescent point source targets: point sources against a transparent background (red circles) and black background (orange circles). We also model three types of extended targets: a black object with or without point sources (black and blue circles, respectively), and a luminous wake (green circles) containing point sources. The black extended object with multiple point sources is intended for modelling of bodies with distributed photophores as well as for stimulated bioluminescence (SB) caused by a black object moving through bioluminescent plankton. The *luminous wake* models the visibility of SB emitting light in the wake after a moving object. Equations for the black object without point sources were not derived separately but modelled as the luminous case with no bioluminescence (*E* = 0).

Detection of a target against a background requires discrimination of signals from two visual channels (pixels), sampling light from the target and the background (figure 1). We can think of these pixels as being retinal areas at least as large as a single photoreceptor, but potentially much larger. We assume that the channels being compared have identical properties. The channels may represent single photoreceptors or larger circular pools of receptors. A target channel detects a mean of *N*_T_ photons per integration time and the corresponding mean count for a background channel is *N*_B_. The photon counts are sums of real photons and intrinsic noise (false photons), and obey Poisson statistics [12,13], where the standard deviation is the square root of the mean. We follow Land [14] and assume a Gaussian distribution of photon samples (this approximation is good when at least one of the two signals to be compared exceeds 10 photon events). Discrimination between the signals in the two channels is possible when the difference is greater than or equal to a reliability constant *R* times the standard deviation of the difference (which is the square root of the sum of the two means [14]): 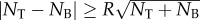. The discrimination threshold is then given by2.1
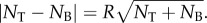

Variables and constants are defined in table 1. By developing expressions for the photon counts, we can now use the criterion to calculate maximum distance at which a target can be detected. This will have to be done separately for each type of detection task.

**Table 1.** Definition of variables (units in brackets). For some variables, we use a typical value (TV) for modelling, if nothing else is indicated.

variable	definition
*N*_T_	mean number of real and false photons detected per integration time in a visual channel aimed at the target (photons)
*N*_B_	mean number of real and false photons detected per integration time in a visual channel viewing the background space-light (photons)
*N*_bio_	mean photon count originating from bioluminescent sources (photons)
*N*_space_	mean photon count from background space-light (photons)
*N*_black_	mean photon count originating from light scattered into the line of sight between target and observer (photons)
*X*_ch_	number of false photons (dark noise) per integration time in a visual channel (photons)
*X*	dark-noise rate per photoreceptor (photons s^–1^), TV = 2.8 × 10^–5^ s^−1^, see [15]
*A*	pupil diameter (m)
*r*	range: maximum visibility distance to target (m)
*E*	number of photons emitted by bioluminescent point source in all directions per second (photons s^–1^)
*I*_space_	radiance of space-light background in the direction of view at the position (depth) of the eye (photons m^–2^ s^–1^ sr^–1^)
*T*	width of extended target (m)
*x*	average distance between point sources across an extended object (m)
*α*	beam attenuation coefficient of seawater (m^–1^), see [5] and the electronic supplementary material
*κ*	attenuation coefficient of background radiance (m^–1^), see [5] and the electronic supplementary material
*λ*	wavelength of light, taken as 480 nm for bioluminescence and transmitted daylight
*n*	refractive index in object and image space, taken as the value of water, 1.33
*d*	photoreceptor diameter (m), TV = 3 µm, see [7]
Δ*t*	integration time (s), TV = 1.16 s [8,16,17]
*q*	detection efficiency: ratio of detected and incident photons, depending on loss in the ocular optics, fraction absorbed in photopigment and transduction efficiency, TV = 0.36 [8]
*f*	focal length (m)
*M*	the ratio of focal length and pupil radius, 2*f*/*A*, here set to Matthiessen's ratio (2.55)
*R*	reliability coefficient, here set to 1.96 for 95% confidence [14]

### (b) Detection of a point source on a transparent target

This case is applicable for detection of single transparent plankton organisms against the background space-light. We assume a pair of visual channels in a camera-type eye. A target channel is aimed at the bioluminescent point source, and its signal is compared to that of a channel aimed at the background next to the point source (figure 1). The target channel is assumed to receive all light that enters the eye from the bioluminescent point source. Because the source does not obscure the background, the target channel receives background space-light of the same intensity as that seen by the background channel. The target channel will receive an average of *N*_bio_ photons per integration time from the point source and *N*_space_ photons from the background space-light, whereas the background channel only receives *N*_space_ photons from the background. Each channel also generates an average of *X*_ch_ false photons per integration time. The total average signal in the target channel will thus be 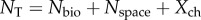 and in the background channel, 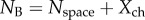. Inserting this into equation (2.1) gives2.2
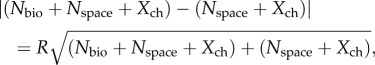
which simplifies to2.3
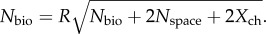


Before we develop *N*_bio_ and *N*_space_, we need to consider the angular size of the two channels. For maximum detection ability, the target channel should collect as much light as possible from the point source, and both channels should collect a minimum of background space-light. This requires that the angular size of the channels be matched to the resolution of the optical image in the eye. Assuming that aberrations are corrected well enough for diffraction-limited optics [18], the optimum visual angle of a channel is limited by diffraction. The main lobe of the diffraction pattern, the Airy disc, contains nearly all the light from a point source, and it spreads from the pupil over an angle of 2.44*λ*/*nA* radians [19], where *λ* is the wavelength of the stimulating light, *n* is the refractive index of water and *A* is the diameter of the pupil (table 1). We know that aquatic camera eyes typically have focal lengths of 2.55 lens radii (Matthiessen's ratio, *M*). Expressed in pupil diameters, the focal length, *f*, is 0.5 *MA*, and the angular diameter of the Airy disc becomes 2.44*λM*/2*nf* radians. If we multiply this by the focal length, we obtain the actual size of the Airy disc on the retina or 2.44*λM*/2*n*. This means that if *M* is constant, the diffraction blur spot has a constant size on the retina irrespective of eye size. For a wavelength of 480 nm and *M* = 2.55, the Airy disc is 1.1 µm wide, and for *M* as high as 3, the Airy disc is still only 1.3 µm wide. But photoreceptor diameters are typically somewhat larger (*d* = 2–6 µm for most rods, cones and rhabdoms), which implies that realistic angular dimensions of the spatial channels should be given by actual receptor diameters rather than any theoretical optimum. We assume a Gaussian profile of the sensitivity across single receptors, where the angular half-width in visual space is *d*/*f* and the solid angle is 1.13(*d*/*f*)^2^, in contrast to the solid angle of *π*/4(*d*/*f*)^2^ for a square angular profile [19].

Ultimately, we are interested in the relationship between the pupil diameter *A* and the range *r*, and search for expressions relating these to *N*_bio_ and *N*_space_. Following Warrant [20], optical geometry gives a photon flux density of *E*/4*π r*^2^ for a point source, attenuation by water is given by 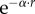 and the pupil area accepting the light is *πA*^2^/4. The product of these factors multiplied by the efficiency *q* of the retina and the integration time Δ*t* provides the desired expression of *N*_bio_:2.4
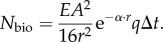


The background space-light is an extended source, and the sensitivity [14] of a retinal channel is simply the product of the pupil area, *π* (*A*/2)^2^, the solid angle in visual space of the channel, 1.13(*d*/*f*)^2^, and the efficiency *q* by which the eye detects photons. We arrive at *N*_space_ by multiplying the sensitivity by the radiance of the background space-light, *I*_space_, and the integration time Δ*t*:2.5
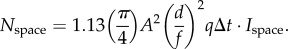
We know from above that 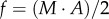, and substituting this into equation (2.5) we obtain2.6
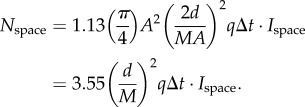
The dark noise per integration time is simply2.7
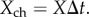


We now insert the developed signal components into equation (2.3), and solve for *A* (for detailed derivation steps, including equations (2.8)–(2.13), see the electronic supplementary material):2.14
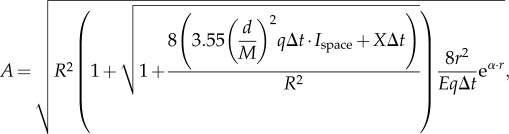


which is the desired relation between *A* and *r* for detection of transparent point sources.

### (c) Detection of a point source on a black target

This case is applicable for detection of single small photophores on the body of a black opaque animal or transparent bioluminescent plankton seen against the black body of an animal (for example with stimulated plankton bioluminescence). Here, we thus assume that the target holding the bioluminescent point source is a black rather than a transparent object (figure 1). For both channels, the target, in this case, interrupts the background space-light, and new light is scattered into the line of sight between the target and the observer. Even a black target will thus contribute light to a visual channel viewing it. The photon count contributed by light scattered into the line of sight, *N*_black_, then replaces *N*_space_ in equation (2.3):2.15
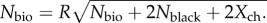


For an observer at constant depth in the sea, space-light enters the line of sight at the rate 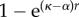, where the attenuation coefficient *κ* depends on the viewing angle [3–5]. For a target at constant depth, the corresponding expression is 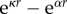, but here we want to determine the space-light at the depth of the observer (from where the eye is performing the discrimination), and thus use the form 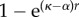. The radiance seen in the direction of a black target then becomes 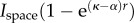. To get an expression for *N*_black_, we substitute 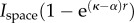 for *I*_space_ in equation (2.6):2.16
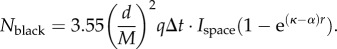


We then repeat the derivation from the previous case and arrive at


2.17
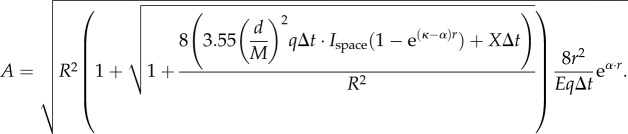


### (d) Detection of an extended black target triggering bioluminescence

This case models the visibility of an animal silhouette. The animal body is assumed to be black, but may contain any number of bioluminescent point sources, either being the animal's own photophores or transparent bioluminescent plankton triggered to emit light by the moving animal. We again assume an equal pair of visual channels, but now optimally sized to detect an extended object against the background space-light. To maximize the signal, the target channel fills the width of the object (figure 1), and both channels have square rather than Gaussian sensitivity profiles (all receptors in the circular pool have equal weight). We thus assume that the angular size of the visual channels (pixel) is dynamic and suited to the object at all times. This detection strategy is chosen because it offers the best detectability with circular pixels (the actual properties of visual channels in pelagic animals is yet unknown). The angle in visual space of such a channel is the target width *T* divided by its distance, *T*/*r* (radians), and with a square profile its solid angle is (*π*/4)(*T*/*r*)^2^ (steradians). Each individual photoreceptor within the circular pool occupies a solid angle of (*π*/4)(*d*/*f*)^2^ in visual space (see table 1 for definition of variables). The number of photoreceptors forming a channel is then (*Tf*/*rd*)^2^ and its diameter on the retina is *Tf*/*r*.

Even though the target itself is assumed to be black, it may contain bioluminescent point sources, but the background is just space-light and no bioluminescence (figure 1). Modelling this way, we are free to investigate both dark (*E* = 0) and luminous extended objects (*E* > 0). The signal of the target channel comes partly from target bioluminescence attenuated on its way to the eye, space-light having entered the line of sight between the target and the eye and dark noise from the contributing photoreceptors: 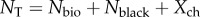, and the background channel sums background space-light and channel noise: 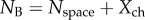.

The discrimination threshold (equation (2.1)) now becomes2.18
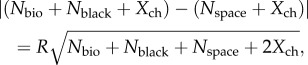
which reduces to2.19
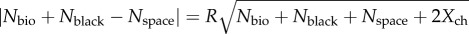
(we here keep the absolute value of the difference because either *N*_T_ or *N*_B_ can have the largest value, depending on the amount of bioluminescence). Note that *N*_bio_, *N*_black_, *N*_space_ and *X*_ch_ are in this case parameters for dynamic receptor pools, and are thus not identical to the same parameters in the point source cases.

We are now ready to work out expressions for *N*_bio_, *N*_black_, *N*_space_ and *X*_ch_, which happens to be easier in the reverse order. The channel noise *X*_ch_ is derived as for the point source case, but here multiplied by the number of photoreceptors in the pool (see above):2.20
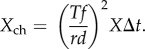
With *f* = *MA*/2, this becomes2.21
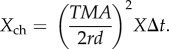
The photon count from background space-light is similar to the point-source case (equation (2.5)) but with *T*/*r* replacing *d*/*f*, and assuming a square rather than a Gaussian profile for the angular sensitivity, we thus replace 1.13 with *π*/4 as follows:2.22
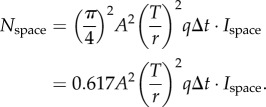


The contribution of light entering the line of sight between the target and the eye, *N*_black_, can be determined by replacing *I*_space_ of equation (2.22) with 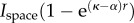 as in equation (2.16):2.23
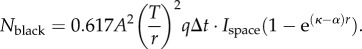


The target may include bioluminescent point sources in the form of photophores on the target animal or planktonic organisms stimulated to emit light by the moving target. The total bioluminescent emission seen by the target pixel, *N*_bio_, can be obtained by multiplying the expression for a single point source (equation (2.4)) with the total number, *P*, of point sources within the field of the target pixel:2.24
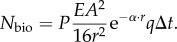


The number of point sources, *P*, seen by the target pixel requires specific expressions depending on the geometric distribution of photophores, or in the case of stimulated planktonic bioluminescence, the expression depends on target motion direction. Different expressions for *P* are given in a separate section after the main derivations and in table 2.

**Table 2.** Expressions for the number of point sources, *P*, seen by a visual channel (pixel) viewing an extended target with distributed photophores, or with plankton causing stimulated bioluminescence (SB).

type of point sources	point source density (per unit area or unit volume)	target area or volume	total number of point sources viewed by the target pixel
photophore array, square packing	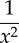	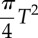	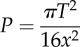
photophore array, hexagonal packing	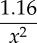	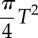	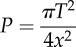
photophore array, random distribution	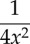	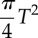	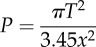
SB, side view of moving target	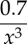	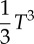	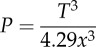
SB, side view of luminous wake	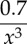	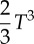	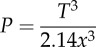
SB, approaching target	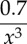	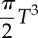	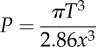

We now substitute equations (2.21)–(2.24) for *X*_ch_, *N*_space_*, N*_black_ and *N*_bio_ in equation (2.19) and solve for *A* (for detailed derivation steps, including equations (2.25)–(2.30), see the electronic supplementary material):


2.31
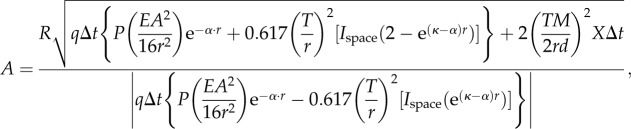


which is the desired relation between *A* and *r* for detection of extended sources. For black objects without bioluminescence, the term 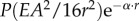 disappears in both the numerator and denominator.

### (e) Detection of an extended transparent target triggering bioluminescence

Finally, we consider the case where the extended target consists of triggered bioluminescence in the wake behind a moving object (figure 1*d*). This is similar to the previous case, except that the target does not block the background space-light. The signal of the target receptor then changes to 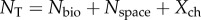, whereas that of the background receptor remains 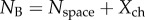. We insert the developed terms as in the previous case and solve for *A* (for detailed derivation steps, including equation (2.32), see the electronic supplementary material):2.33
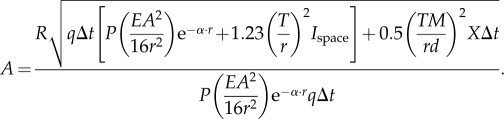
This expression provides the desired relation between *A* and *r*.

### (f) Expressions for the number of bioluminescent point sources in extended targets

A straightforward method to determine the number of photophores, *P*, on a target animal that is seen by the target pixel is to multiply the density of point sources (per unit area) with the area seen by the target pixel, (*π*/4)*T*^2^. For regular square arrays, the density is simply 1/*x*^2^, where *x* is the distance between neighbouring photophores. Hexagonal arrays have a slightly higher density of 1.16/*x*^2^ [21]. For random distributions, the density is 1/4*x*^2^ [21], and here *x* is the average distance to the nearest neighbour. The best expression for *P* thus depends on how the photophores are arranged on the side of the target facing the observer.

The situation is rather different if the bioluminescence is caused by plankton disturbed by the moving target animal. We here assume that the disturbed water volume can be estimated from that displaced by the moving target. The number of point sources, *P*, in this case is the part of the disturbed water volume seen by the target pixel multiplied by the density of bioluminescent plankton in the water (here per unit volume). With an average nearest-neighbour distance, *x*, in three dimensions, the density is 0.7/*x*^3^ [22]. For a target animal that moves perpendicular to the line of sight of the observer, we assume that the disturbed water volume is a cylinder with the same diameter as the moving animal. The target pixel, which also has the diameter of the target, thus defines an observed volume, which can be approximated by the intersection of two perpendicular cylinders, known as a Steinmetz solid (the visual field of the pixel is in fact a cone, but at sufficient target distances a cylinder is a good approximation). The target body obscures half this volume. With the target diameter, *T*, this volume is (1/3)*T*^3^ [23]. If the plankton continues to glow long enough, there will be a luminous wake behind the animal, and with the body out of the way, the full volume, (2/3)*T*^3^, will be visible to the target pixel. The disturbed water volume created by a target moving straight towards or away from the observer can be estimated as a cylinder of diameter *T* along the line of sight. Here, we assume that the unobscured length of this cylinder is 2*T*. The observed volume is then (*π*/2)*T*^3^. The different cases and expressions of *P* are summarized in table 2.

## 3. Results and discussion

The maximum distance at which objects can be visually detected is an important ecological factor because it shapes foraging, and prey/predator relations as well as interactions with conspecifics [24]. The theoretical framework developed here to investigate vision in pelagic habitats allows us to investigate general principles of visual ecology in this largely inaccessible habitat. In analogy with cameras and telescopes, visual performance is limited primarily by the physics of light. From this, it follows that eye size, or more specifically the pupil diameter, sets very sharp performance limits on the vision of all animals. Knowing these performance limits is a prerequisite for understanding both the roles of vision and the degrees of investment in vision made by different animal groups and species. To exploit all the possibilities opened up by the theoretical tools developed here, it would require comparisons with much of the existing knowledge on pelagic ecology in a wide range of animals. This would be a massive undertaking, far beyond the scope of this paper. Here, we restrict the discussion to short descriptions of some questions where we believe that the theory may provide valuable insight into pelagic visual ecology.

### (a) Types of targets

**Figure 2.**
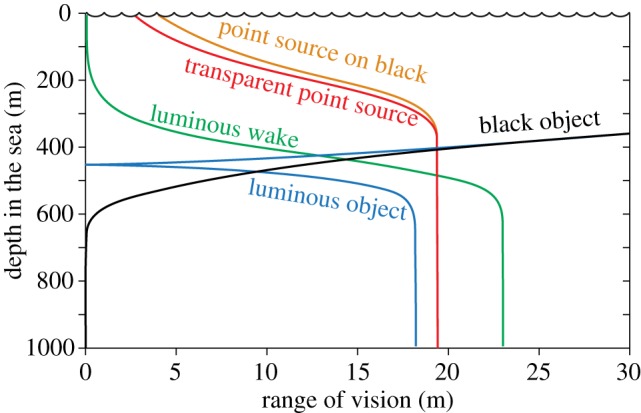
The maximum detection distance (visual range) for different types of targets, plotted as a function of depth in the sea (on a sunny day). For this type of graph, we consistently use an unconventional orientation with the dependent variable on the horizontal axis, in order to represent water depth, which is a vertical property, on the vertical axis. Modelling conditions: maximally clear oceanic water (blue water, Jerlov type I [25]), sun at 45° (see the electronic supplementary material), horizontal viewing, 10 mm pupil diameter, 0.5 m target width, 10^10^ quanta s^−1^ point source intensity. For a luminous object and a luminous wake, we assume a side view and an average nearest-neighbour distance of 0.3 m between bioluminescent plankton (table 2). The luminous object is assumed to have point sources distributed on a black background. At depths below 600 m, it is visible up to a distance of 18 m, and there is practically no competition by background space-light. Above 600 m depth, the increasing space-light shortens the detection distance, and at about 430 m depth the background radiance exactly matches that of the target such that it becomes invisible (zero detection distance). At depths above this cusp on the curve, the luminous object appears darker than the background, and above 400 m depth, the luminous object curve joins that of a black non-luminous object (the modelled bioluminescence is here insignificant in relation to downwelling daylight).

Because downwelling daylight gradually becomes dimmer with depth, non-luminous objects are obviously best seen close to the surface, whereas bioluminescent objects are more visible in deep waters. A comparison of the visual range for detecting point-light sources, black silhouettes and extended luminous sources is illustrated in figure 2. Even though the functions depend on water quality, viewing angle, pupil diameter and the width of extended objects, the general features are similar under all conditions. In shallow water, large dark silhouettes offer by far the longest visual range, but below a certain depth they cannot be detected at all. Bioluminescent point sources can be seen surprisingly well even at shallow depths, and it does not matter much if they are seated on black or transparent objects. Below a rather well-defined depth, the competition with daylight ceases such that the visual range of point sources remains constant at increasing depth. Extended luminous objects, both black and transparent (luminous wake), compete with daylight much deeper in the ocean than point sources do. They are also much less visible in shallow water. A black silhouette covered with bioluminescent point sources behaves as a non-luminous object in shallow water, and as a luminous object in the deep. At an intermediate depth, the bioluminescence exactly corresponds to the space-light radiance, making the object perfectly camouflaged (here occurring at a depth of about 460 m). This type of diagram can provide valuable information on the types of objects that are most or least visible at different depths, and thus suggests the visual strategy of choice for particular species.

### (b) Eye size and visual performance

**Figure 3.**
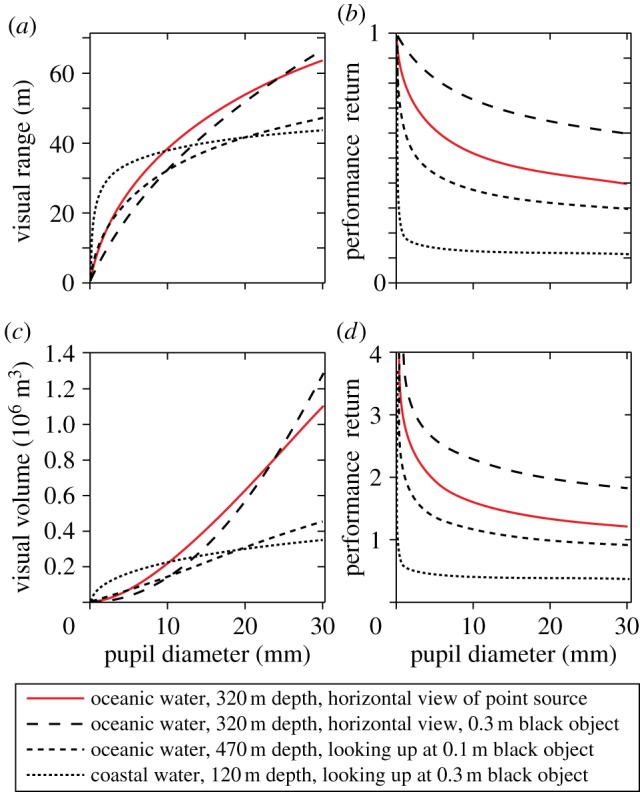
Visual performance as a function of eye size (pupil diameter). The visual ranges for four different targets are plotted in (*a*), and the corresponding performance returns (d*r*/d*A*) are plotted in (*b*). The same calculations as in (*a*,*b*) but with the visual range converted to visual volume, is given in (*c*,*d*). Point source intensity: 10^11^ quanta s^−1^. The coastal water type is comparatively clear and most closely corresponds to Jerlov type III [25]. See the electronic supplementary material for water quality and radiance versus depth.

An interesting outcome of modelling the relationship between pupil diameter (eye size) and visual range is that the performance returns of having a larger eye are lower the larger the eye is to start with (figure 3). This law of diminishing returns is inescapable and it results from absorption and scattering of light in the water [26]. Consequently, growing eyes large is much more rewarding in clear ocean water than in murky coastal water. Animals living in turbid water would thus be expected to have comparatively small eyes.

The law of diminishing returns depends quite significantly on the type of target, and also on depth and viewing angle (figure 3*a*). This can be seen as different declines of the slope of the curves, and more directly by plotting the performance return (figure 3*b*), which is the relative increase in visual range for a small increase in eye size. If the visual range increases by 0.5% as a result of a 1% increase in eye diameter, the performance return is 0.5. For very small eyes, the performance return is 1, but it drops significantly already at a pupil diameter of just a few mm.

For detection of objects that can appear in any direction, the total monitored water volume is probably more directly related to fitness than visual range is. Because the monitored water volume has a cubic relation to visual range, it pays much more to increase the eye size (figure 3*c*,*d*) with this measure of performance. The lesson is that species using vision primarily to scan their environment for detecting objects occurring in random directions should have relatively larger eyes compared with those that use vision mainly for pursuit or recognition tasks. This may explain some of the large variations in relative eye size reported in myctophid fish [27].

**Figure 4.**
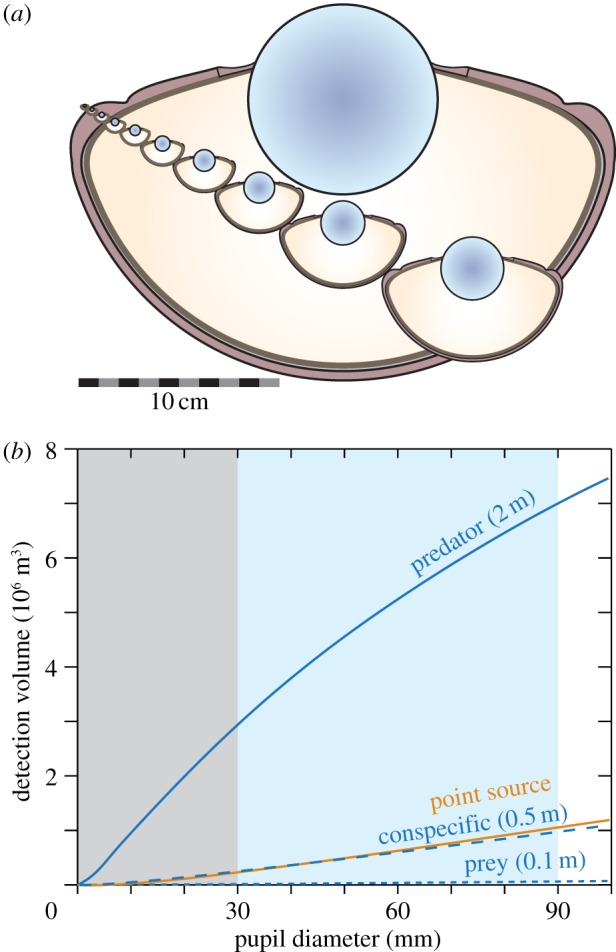
The benefit of huge eyes in giant and colossal squid. An illustration of eye size (*a*) shows the continuous range of eye size in fish, cephalopods and whales, up to that of swordfish (3 cm pupil diameter [28]), and the leap up to 9 cm pupil diameter in giant and colossal squid [10]. Visual volume as a function of pupil diameter (*b*), calculated for stimulated bioluminescence below 800 m depth where there is no influence of daylight and performance is independent of viewing direction (from [11]). The ability to detect very large luminous objects (2 m wide) is superior and grows steadily for eye sized up to those of adult giant and colossal squid. The shaded areas indicate the range of eye sizes found in animals except giant and colossal squid (grey), and the range unique to these large cephalopods (light blue). Modelling values (from [11]): clear oceanic water, 10^10^ quanta s^−1^ point source intensity, 0.55 m nearest-neighbour distance between point sources, approaching target triggering a water volume calculated as a cylinder of target width (0.1 m, 0.5 m, 2 m) and a length of 2.5 target widths, receptor diameter 0.4 µm.

An interesting question is why giant and colossal squid have eyes that grow to about three times the diameter of the eyes of any other species, including large fish and whales (figure 4*a*). For most visual tasks under water, the law of diminishing returns would act against investment in such huge eyes. In two separate studies [10,11], we analysed this question and found that detection of stimulated bioluminescence caused by very large objects (about 2 m wide) provides a unique motivation for very large eyes (figure 4*b*), and it was suggested that early detection of foraging sperm whales at depths below 800 m might have generated particularly strong selection for huge eyes in these two species of squid.

### (c) Sighting range of black targets

**Figure 5.**
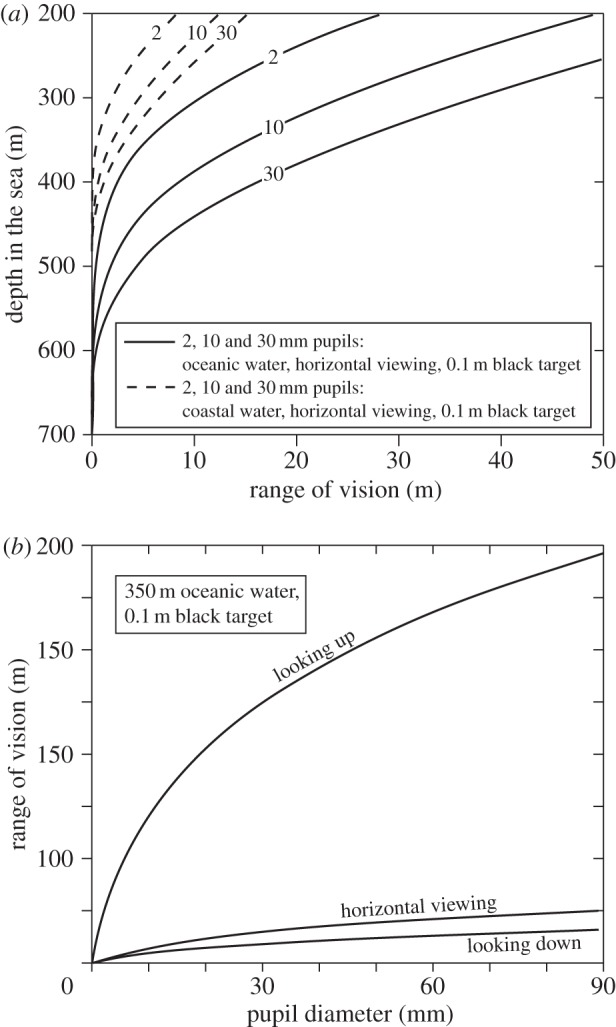
Visual range of black extended targets (with no bioluminescence). In (*a*), range is plotted for different depths, for different pupil diameters and different water qualities. In (*b*), range is plotted against pupil diameter for different viewing directions. See the electronic supplementary material for water quality and radiance versus depth.

Especially in fresh-water bodies, visibility is regularly measured as the depth where a standardized Secchi disc is just visible [29,30], and divers often refer to a specific visibility distance associated with the water quality. In reality, the water quality does not determine a fixed distance beyond which vision does not reach. The amount of daylight, the size and contrast of the target, the pupil diameter and the viewing angle are all factors determining the range of vision (figure 5*a*,*b*). Very large ships seen from below in clear oceanic water would offer the longest visibility distance of non-luminous objects in the pelagic. But the optical water quality is an important factor that makes the conditions for vision in oceanic, coastal or estuarine habitats very different. In clear oceanic water, even small eyes can see black objects at considerable distance, but in less clear coastal water the visual ranges are much shorter even for very large eyes (figure 5*a*). The viewing angle also has a dramatic effect on visual range (figure 5*b*). Dark silhouettes are seen at very much longer distances looking upwards compared with looking horizontally or downwards [5,31]. This is the obvious reason why so many mesopelagic animals have tubular eyes for spotting prey above [15]. It is also the reason for counter-illumination, which offers the only way to conceal the silhouette [31]. In habitats with clear water and dense populations of animals, for example the epipelagic zone of tropical seas close to coral reefs, even a relatively small eye provides a visual range that may substantially exceed the animal's range of action or range of interest. In these habitats, investment in relatively large eyes should be rare.

### (d) Depth limits for vision using the sun, the moon or starlight

**Figure 6.**
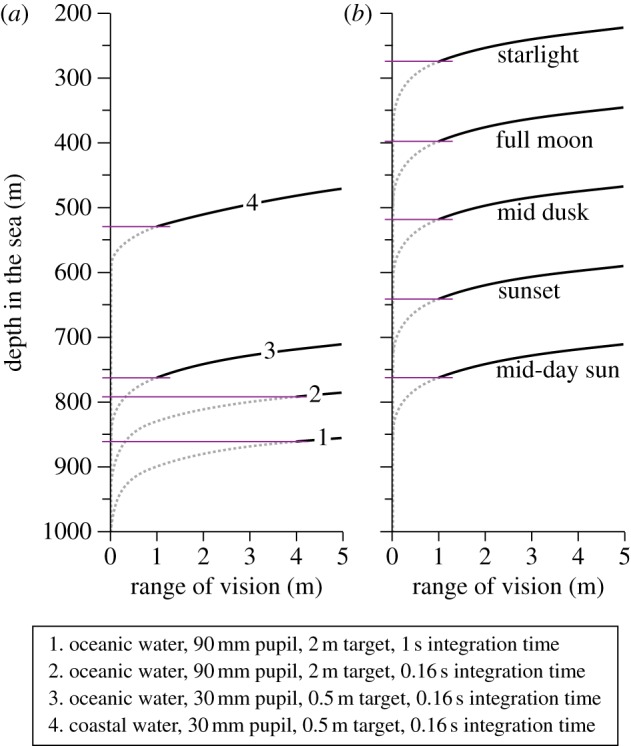
The depth range for upward detection of non-bioluminescent targets. Horizontal lines indicate the depth at which the visual range equals twice the target width (see main text). Different pupil diameters and integration times for bright downwelling sunlight are compared in (*a*), and curve 3 is recalculated for different terrestrial luminance conditions in (*b*). See the electronic supplementary material for water quality and radiance versus depth.

The maximum depth at which daylight can be used for vision is typically suggested to be between 700 and 1000 m for clear oceanic water [20,31–33]. Our computational approach offers a way to find the theoretical depth limit for vision in downwelling daylight. A large black target, seen from below, allows the longest sighting range possible for non-luminous targets. Using a biologically realistic maximum target-width of 0.5 m, the visual range for an eye with 30 mm pupil diameter approaches zero at about 850 m in clear oceanic water (figure 6*a*, curve 3). But this is a significant overestimate of actual limits, because at very close range, the target occupies a very large visual angle (close to 180°), and thus totally occludes the background it is supposed to stand out against. This is assuming a purely spatial signal. If a large object suddenly gets very close, there will of course be a temporal signal (i.e. a darkening) that the animal can detect without any spatial comparison.

To give room for two adjacent visual channels (pixels) to compare a target with a background, we assume that the target must not come closer than, say, twice its own width (covering 28°). With this assumption, the maximum depth shrinks from 850 to 760 m (figure 6*a*). In coastal water, where the downwelling radiance drops more rapidly with depth, the daylight vision zone ends considerably further up in the water column. At sunset, dusk and night, the depths at which downwelling light can be used for vision is of course greatly reduced (figure 6*b*). Surprisingly, even starlight may be sufficient for some visual tasks at depths exceeding 200 m.

Because a large pupil diameter allows vision at lower intensities, it is of interest to calculate the depth at which giant and colossal squid can use their enormous eyes with 90 mm pupil diameter. It turns out that the limit is between 790 and 860 m depending on the integration time (160 ms and 1 s: figure 6*a*). It is important to note that these depths are calculated for the clearest ocean water at midday in the tropics. Everywhere else, the maximum depth for vision in downwelling daylight will be much less [34].

### (e) Prey/predator relations

**Figure 7.**
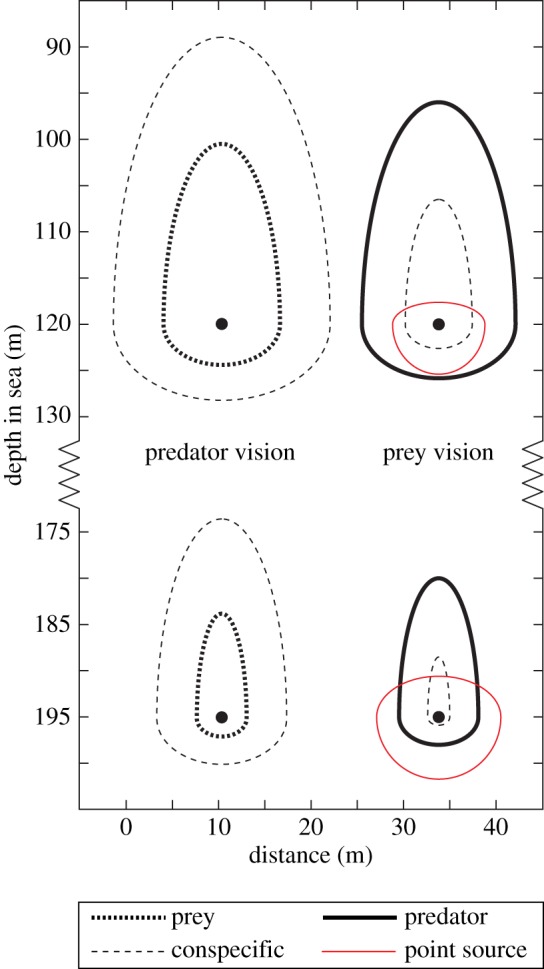
Visual range of a hypothetical prey/predator pair plotted as visibility bubbles at two different depths (120 and 195 m in coastal water during the day) with visual range at the same scale on both axes. The vertical axis also gives the actual depth in the sea. The predator is assumed to have an 8 mm pupil diameter and is viewing prey animals (which are 1 cm wide) and conspecifics (which are 10 cm wide). The prey is modelled with a pupil diameter of 2 mm viewing 10 cm wide predators, and 1 cm wide conspecifics as well as bioluminescent point sources emitting 10^11^ quanta s^−1^.

In terms of vision, a pelagic animal is surrounded by a volume of water beyond which vision is not possible. This visibility ‘bubble’ follows the animal as it moves, and visual interactions with other animals (prey, predators or conspecifics) can take place only within this bubble. Obviously, the bubble depends on pupil diameter and varies with the type of and size of target (figure 7). A large predator may have a large pupil area, but it will typically be searching for smaller prey. The prey, in contrast, has smaller pupils but will be looking out for a larger predator. Which of them has the largest visibility bubble will depend on eye sizes and body sizes, but an interesting observation is that the relative width and heights of predator/prey visibility bubbles do not change dramatically with depth (figure 7). That is because the radiance field assumes a relatively constant form for depths below about 200 m [1]. Another observation is that the diameters of the visibility bubbles in clear oceanic water are often one to two orders of magnitude larger than the body lengths of animals. In murky coastal or estuarine water, exhibiting much more absorption and scattering [25], the diameters of the visibility bubbles are instead in the same order of magnitude as body length. This, of course, implies that visual ecology is fundamentally different in different water types, and our theoretical framework provides a way to reveal some of these differences.

### (f) Counter-illumination

**Figure 8.**
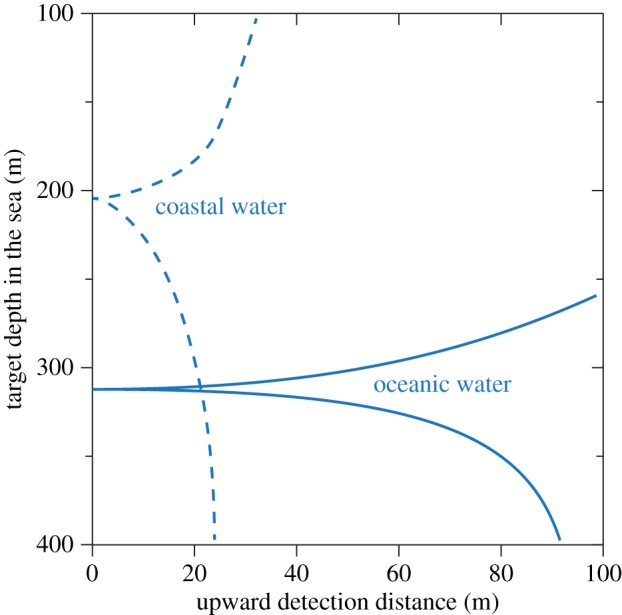
The visual range for counter-illumination against the downwelling daylight. The sharp cusps on the functions are at the counter-illumination depth, at which the target is invisible from below (zero visibility range). Above this depth, the target will appear dark on a brighter background, and below this depth the contrast is reversed. To remain invisible, the counter-illuminating animal will either have to adjust its depth according to its bioluminescent intensity or to dynamically control bioluminescence to match the downwelling radiance at different depths. Modelling data are from [38], for a counter-illuminating shark.

Many pelagic animals (fish, squid and crustaceans) use ventral photophores to camouflage their otherwise prominent dark silhouette [15,35–37]. The principle is that bioluminescence replaces the light blocked by the body. This is an important strategy because, as we have seen earlier, a predator looking straight up can see a dark prey silhouette from a very long distance, whereas the prey has great difficulty in seeing what is approaching from below. But intensity matching is important if the camouflage is to be effective. It is well known that any angular or spectral mismatch in the emitted light will hamper the camouflage [35,36]. Our modelling (figure 8) reveals how extreme the precision must be, and how severe the consequences are of even a slight vertical displacement. Especially in clear ocean water, an animal becomes visible at very long distances if it is only a few metres shallower or deeper than the isoluminance depth (figure 8). In effect, this means that counter-illumination is not a simple and foolproof way to become invisible in the pelagic. It also suggests that mechanisms for precise intensity matching are essential, such as the bioluminescence control in hatchetfish [39] and the opsin expression in squid photophores [40]. The shorter detection ranges of less clear water makes the situation a little more forgiving.

### (g) Resolving individual point sources or pooling many

For maximal visibility of point-light sources, the visual channels should cover as narrow an angle as possible. This way, the signal will be minimally contaminated with background space-light. But resolution of single point sources is not necessarily the best strategy if the target consists of multiple photophores or is a moving object stimulating bioluminescence from many planktonic organisms. With low resolution (extended-source viewing with large pixels), it is possible to sum up many point sources into the same visual channel, and thus obtain discrimination at longer distances than a single point source would allow (analogous to spatial pooling for improving vision in conventional extended scenes in dim light [8]). The large pixels will of course pick up more space-light, although this is a problem only when the background is bright. The question whether extended-source viewing or point source detection is the best strategy will depend how many point sources the larger pixels will see. In dark environments, extended source viewing will generally be superior if the pixels detect an average of more than one point source. Detection of many point sources in each target pixel can lead to much longer sighting distances compared with detection of individual point sources.

**Figure 9.**
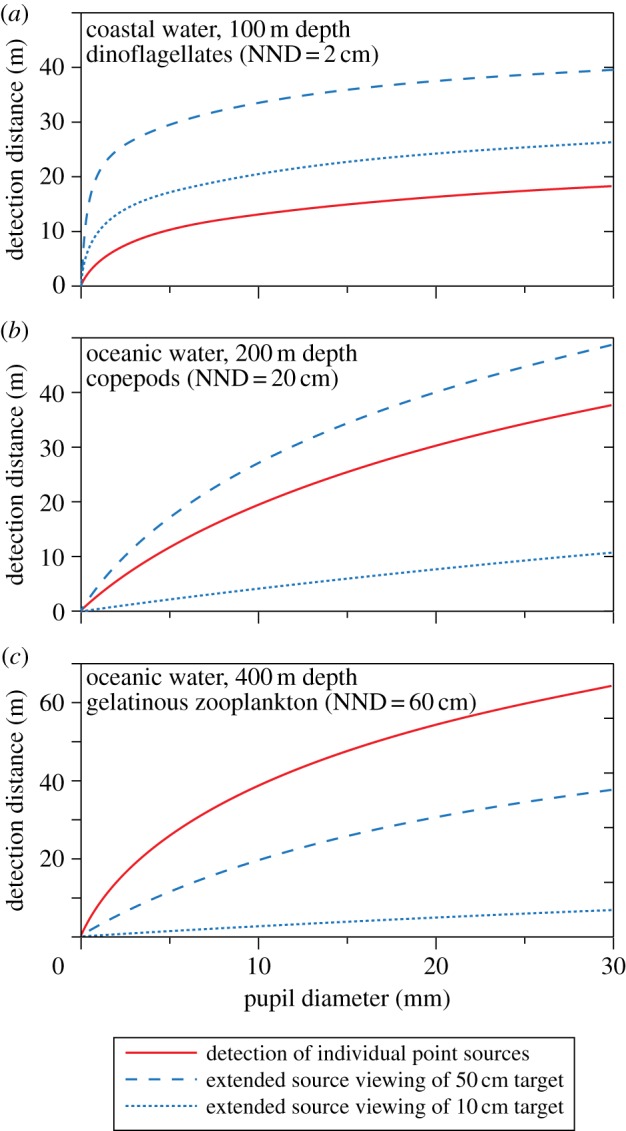
Comparison of extended source and point source viewing at night (starlight) for horizontal detection of stimulated bioluminescence of different point-source intensities and nearest-neighbour distances (NND). The three panels are calculated for assumed conditions of bioluminescence from dinoflagellates (*a*), copepods (*b*) and gelatinous zooplankton (*c*). Point source emission was assumed to be 10^11^ quanta s^−1^ for dinoflagellates and gelatinous zooplankton, and 10^10^ quanta s^−1^ for copepods. Extended sources are calculated as side views of a moving black target (table 2).

During dinoflagellate blooms in shallow water, there may be in excess of 100 cells per litre [41]. Under these conditions, a moving fish will trigger so many flashes that extended-source viewing makes for a longer visual range even for target widths below 10 cm (figure 9). But with common densities of gelatinous zooplankton in deep water (0.5 m nearest-neighbour distance [42]), detection of individual point sources is superior unless the moving target is very large. Similar considerations apply to ventral photophores, where the pattern of photophores may be resolved at close range by conspecifics, but matched to the background and thus invisible to predators at longer range [43].

### (h) Sensitivity to physiological parameters

**Figure 10.**
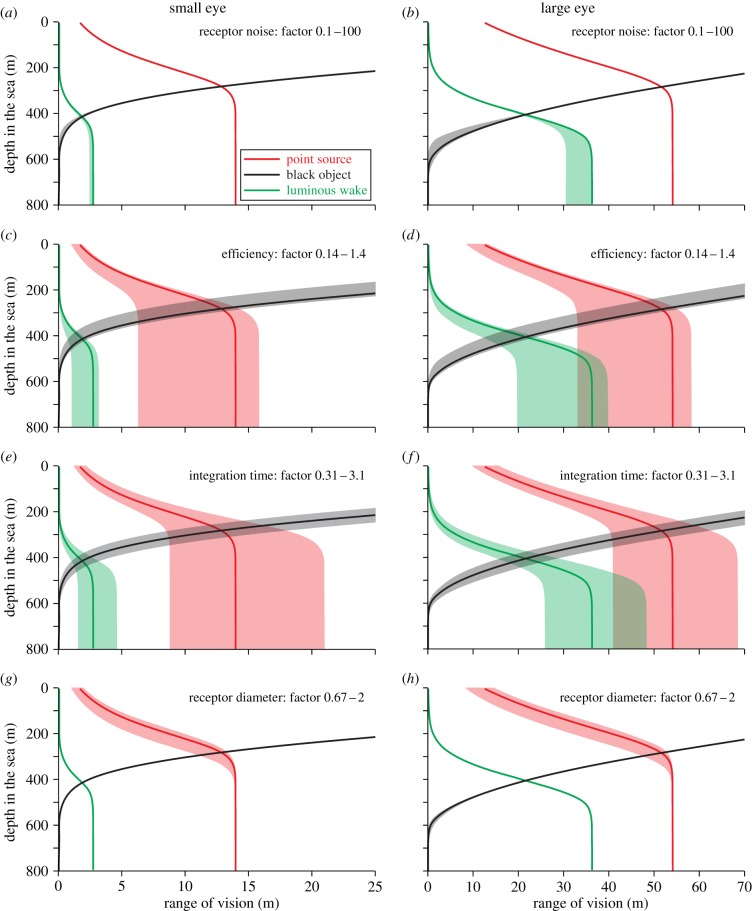
The effect of variations in (*a*,*b*) receptor noise, (*c*,*d*) quantum efficiency, (*e*,*f*) integration time and (*g*,*h*) receptor diameter. The solid lines are calculated for the typical values indicated in table 1, and the shaded areas indicate the consequence of decreasing and increasing the values by the factors indicated in each panel. The left column of panels is calculated for a small eye (pupil diameter 2 mm) and the right column for a large eye (pupil diameter 20 mm). Calculations are for daytime horizontal viewing in oceanic water, the point source intensity is 10^11^ quanta s^−1^ and 0.3 m NND (SB side view, see table 2), and the extended target width is 0.3 m wide.

Our calculations are based on a large number of assumed values. In reality, these values may vary between species and assumed typical values may not be representative in all cases. By testing the sensitivity to variations in parameters, such as receptor dark-noise, quantum efficiency, integration time and receptor diameter, it is possible to assess the reliability of our modelling. But physiological parameters that are critical to our modelling are also critical to the animals. We can thus use the computational approach to learn how selection is likely to act on some key physiological parameters (figure 10).

Varying receptor noise over three orders of magnitude has surprisingly little effect on visual performance (figure 10*a*,*b*). It is only in the dim light below 400 m that receptor noise has any notable effect. The reason for this is that receptor noise in vertebrate rods and invertebrate rhabdoms is extremely low [44]. For extended sources, the impact of receptor noise grows with eye size, simply because there are more photoreceptors per unit angle in visual space. Retinal efficiency (the fraction of incident photons that are detected) and integration time have similar and very large effects on visual performance (figure 10*c–f*), especially under dim conditions. This means that calculations of visual performance in specific animal species should be interpreted with caution unless these parameters can be accurately estimated. It also points to a need for measurements of quantum efficiency and integration time in pelagic animals. The strong effect on performance suggests that quantum efficiency and integration time could be under strong selection, and this is probably true for efficiency but with integration time there is of course the standard trade-off with temporal resolution [8], which is not considered in our theory. The receptor diameter (figure 10*g*,*h*) has an impact mainly on the detection of point sources against a background of space-light. Although not the subject of this study, the receptor diameter may also be important for resolution of fine patterns. We also investigated the consequences of varying the F-number between 2.5 and 3.5, but found it to have only negligible effects (not illustrated).

### (i) Spatial sampling

For point source detection, we have assumed that the visual system can use the full resolution of the retina and for extended sources we have assumed that receptor pooling can adjust dynamically to the target. Neither of these assumptions can be expected to hold entirely for all species, or for all parts of their retinas. To feed the brain with one axon for each photoreceptor is not possible unless the eye is tiny. But it is possible that single-channel detections can be summed onto a smaller number of neurons in early processing. This need not affect the visual range but it will reduce the number of pixels, and the possibility to resolve dense patterns of point sources. The dynamic spatial summation assumed for extended-source vision is a well-known phenomenon in visual systems [8]. But this does not mean we would expect summation dynamics to operate without limits. It is more likely that selection has shaped the visual system of each species such that spatial summation can be adjusted to cope with naturally occurring and biologically meaningful stimuli. For these reasons, individual species may come close to some of the theoretical performance limits, but not to others. But it would be surprising if evolution has failed to exploit the potential relevant to the life style of each species.

### (j) Outlook

A framework for assessing visual performance is a versatile tool for investigating general principles of visual ecology. The examples given in this discussion only superficially touch on a few of the questions that can be analysed using the equations derived here. The theory has the potential to explain allometric relationships between eye size and body size, and to investigate relationships within and between species. An interesting and little understood area is the visual ecology of turbid river habitats (or algal blooms) where vision acts on completely different scales compared with vision in clear water. Vertical migrations, other than those that just follow isolumes, offer another area where the computational approach may help to reveal general principles. However, for accurate modelling of vision in the upper 100–200 m of oceanic water, where light is not effectively monochromatic [25,31], it is necessary to use more elaborate calculations of the daylight intensity available for absorption in a photoreceptor, *I*_space_ (see the electronic supplementary material, S2: Modelling of radiance and absorption coefficients in the sea).

The theory developed here can also be expanded and elaborated. An obvious extension is to cover white or grey targets. This would allow analyses of the consequences of body coloration. It is also possible to develop expressions for pelagic vision at all angles (not just vertical and horizontal as in this paper), and to take the variation in sensitivity over the visual field into account. With such additions, it would be possible to assess in more detail the visual information available to different species. An urgent extension of the theory is of course to modify the expressions for other types of eyes, for example compound eyes. In fact, the theory is already valid for compound eyes of the superposition type, but not for apposition eyes.

An alternative to modelling visual range is to calculate spatial resolution, which would open the possibility of analysing most visual tasks that are not based on simple object discrimination against a homogeneous background. Such a development is particularly relevant for analysis of benthic and terrestrial habitats. Extensions to allow analysis of colour and polarization contrasts are also desirable. Taken together, computational visual ecology is a powerful tool that can inform us about how well different visual tasks can be performed under given circumstances, and thus how the adaptive landscape of vision actually appears.
